# Lower Extremity Marjolin’s Ulcer Reconstruction With Free Anterolateral Thigh Flap: A Case Series of 11 Patients

**DOI:** 10.7759/cureus.11392

**Published:** 2020-11-09

**Authors:** Shobhit Sharma, Nikhil Das, Vivek Gupta, Sudipta Bera, Navneeta Bisht

**Affiliations:** 1 Surgery, Shri Ram Murti Smarak Institute of Medical Sciences, Bareilly, IND; 2 Surgery, Sapthagiri Institute of Medical Sciences and Research Centre, Bengaluru, IND; 3 Plastic and Reconstructive Surgery, Sir Ganga Ram Hospital, New Delhi, IND; 4 Plastic and Reconstructive Surgery, Institute of Medical Sciences, Banaras Hindu University, Varanasi, IND; 5 Anaesthesiology, Shri Ram Murti Smarak Institute of Medical Sciences, Bareilly, IND

**Keywords:** lower extremity, flow-through flap, reconstruction, anterolateral thigh (alt) flap, marjolin’s ulcer, free alt flap

## Abstract

Background

Marjolin’s ulcer (MU) of lower extremities usually presents with scar contracture and functional disability. They often follow an aggressive course and poor outcome, and require early radical removal. Split-thickness skin grafts, local flaps, or amputation are commonly practiced surgical options for MU. Though free flaps are gaining popularity for various oncoplastic reconstruction, they are not frequently used for MU. A free anterolateral thigh (ALT) flap may have a beneficial role as it provides simultaneous coverage for a large defect after radical tumor and scar excision.

Methods

Between January 2015 and December 2018, 11 patients with lower limb MU reconstructed with free ALT flap were reviewed retrospectively for the surgical procedure, recurrences, and functional outcomes.

Results

Mean dimensions of the defect and flaps were 8 cm × 6 cm and 18.91 cm × 11 cm, respectively, and total flap coverage was obtained in nine cases. Marginal flap loss was noted in one and residual contracture in two cases. Functional improvement of the limb was achieved in all cases. Recurrence or disease-related mortality was not seen in any patient after a mean follow-up of 35.82 months.

Conclusions

Free ALT flap reconstruction of MU of extremity facilitates most radical tumor and scar-contracture removal and thus reduces the chances of re-ulceration. It facilitates local radiotherapy protocol with the provision of immediate durable coverage. Thus, it has a beneficial role other than a secondary reconstructive procedure. Moreover, an added benefit may be obtained with a “flow-through’ flap” to avoid amputation and improve functional outcomes.

## Introduction

Marjolin’s ulcer (MU) is an infrequently encountered malignancy, occurring in a previous scar and commonly seen in the lower extremity. Post-burn scar or non-healing ulcers on lower extremity are particularly susceptible to MU as they are subjected to continuous stretching during daily movement [1,2]. MUs usually present with squamous cell carcinoma (SCC) in the milieu of a scar and deep-seated contracture, restriction of major joint movement, and functional disability. The outcome of extremity MU is negatively implicated due to a high chance of advanced disease, aggressive course, recurrence, functional loss, and amputations [2-6].

Wide local excision (WLE) and resurfacing with split-thickness skin graft (STSG), local flaps, or amputation are commonly practiced for the surgical management of extremity MU. The particular concern for MU surgery is the amount of tissue resection. By convention, a 2- to 5-cm margin clearance is recommended for MU [7-9]. But for extremity MU, plane and margin of resection are often not very evident due to the presence of concomitant deep-seated scar, contracture, and three-dimensional tissue involvement. Simultaneous contracture release and complete removal of the potential scar necessitates a greater amount of tissue to resurface the defect. Residual scar tissue leads to the persistent functional disability and the chance of re-ulceration. On the other hand, aggressive resection leads to the exposure of vital structures and may lead to an amputation. A free flap may have a beneficial role as it addresses simultaneous contracture release with radical scar excision and resurfacing of exposed vital structures.

We present here a series of 11 cases of lower extremity MU resurfaced with free anterolateral thigh (ALT) flaps and their outcome analysis over the last five years.

## Materials and methods

All patients with post-burn lower extremity MU reconstructed with free ALT flap between January 2015 and December 2019 were reviewed retrospectively in this study. The study was conducted after clearance from the Institutional Ethical Board and as per standard ethical guidelines. Informed written consent was obtained from each patient for the utilization of the data for the study purpose. Patient-related variables were collected from the hospital database.

Patients attending the Outpatient Department with a history of ulcer or lesion over a long-standing scar, a recurrent ulcer on previous scar tissue, or a non-healing ulcer of an old burn injury of the lower extremity were identified. A wedge biopsy of the lesion was performed to confirm the diagnosis of MU. The dimension of the lesion was noted, and regional lymph nodes (LNs) were assessed clinically. The degree of contracture was noted in case of major joint involvement. Pre-operative MRI of the lesion was performed to note the deeper extent of the lesion. Written informed consent for surgery was obtained from patients and attendants as per Institutional Protocol. WLE of the lesion with at least 2-cm margin with simultaneous contracture release by full-thickness excision of the scar was performed. The scar tissue was excised radically till the complete release of contracture or encountering neurovascular structure, bone, or joint. Margins were assessed with frozen section biopsy and re-excised if required before final reconstruction. All the defects were resurfaced with free ALT flap from the opposite limb irrespective of the dimension. Removal of the involved segment of vessels was performed and considered for a ‘flow-through’ ALT flap reconstruction in case of perivascular involvement. The flap donor site and the residual defect was skin grafted. Regional LN sampling or dissection was performed whenever palpable.

Post-operative radiotherapy was given in larger lesions (>10 cm), in margin positive cases after a re-excision, and high-grade tumor on histopathological examination (HPE). Patients were followed up with a periodic assessment of recurrence and functional recovery. Suspicious lesions were sent for HPE. Ranges of active and passive joint movements, residual re-contracture, and limb length discrepancy were evaluated.

All results were tabulated, and statistical analysis was performed using GraphPad Prism version 8.0.0 software (GraphPad Software, San Diego, CA, USA).

## Results

**Figure 1:**
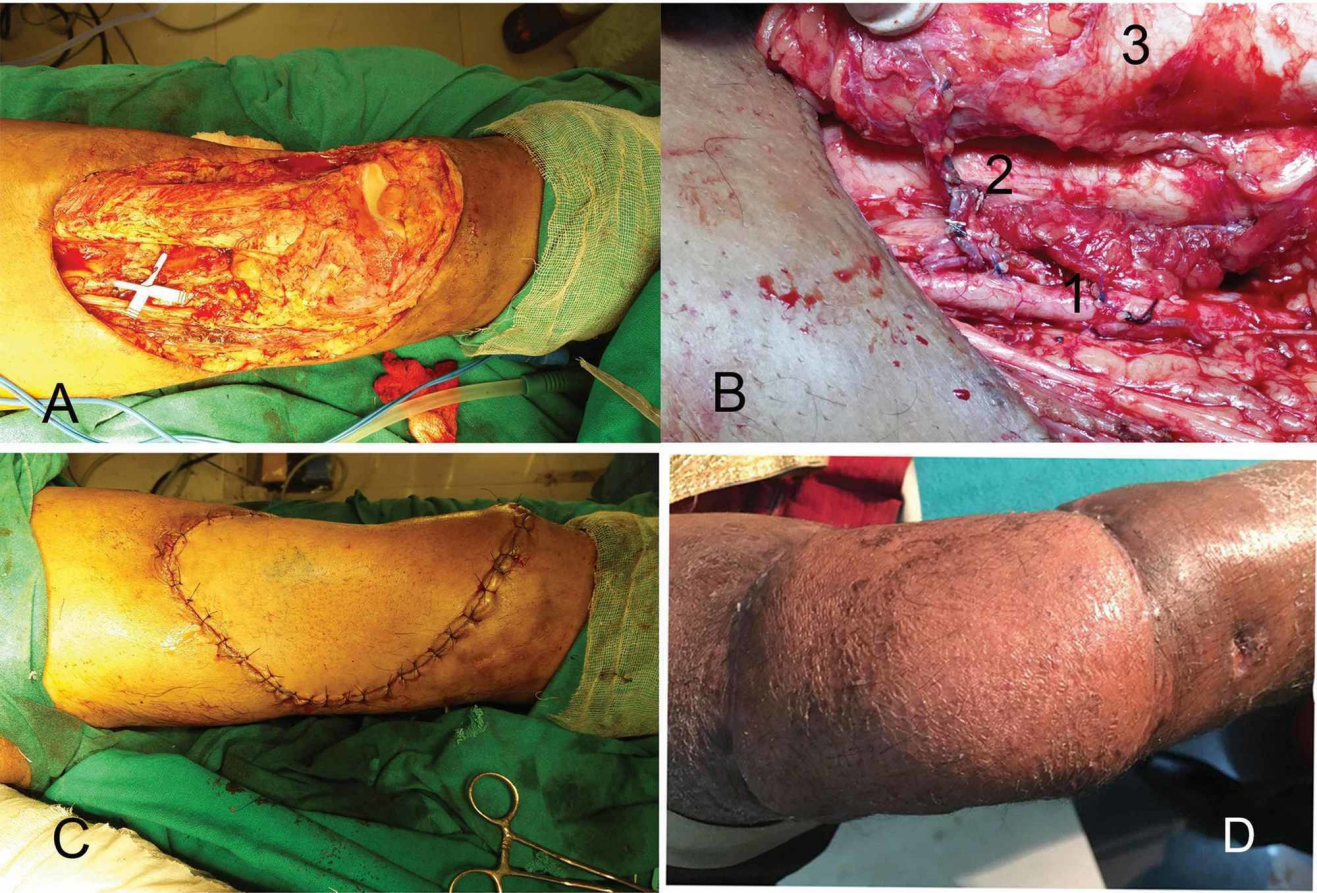
Case 7: Marjolin’s ulcer of thigh demonstrating microvascular anastomosis. (A) Wide local excision defect with exposed femur and knee joint. (B) Microvascular anastomosis (1, femoral artery; 2, lateral circumflex femoral artery perforator). (C) Flap inset. (D) Three-year follow-up showing a well-settled flap.

**Figure 2:**
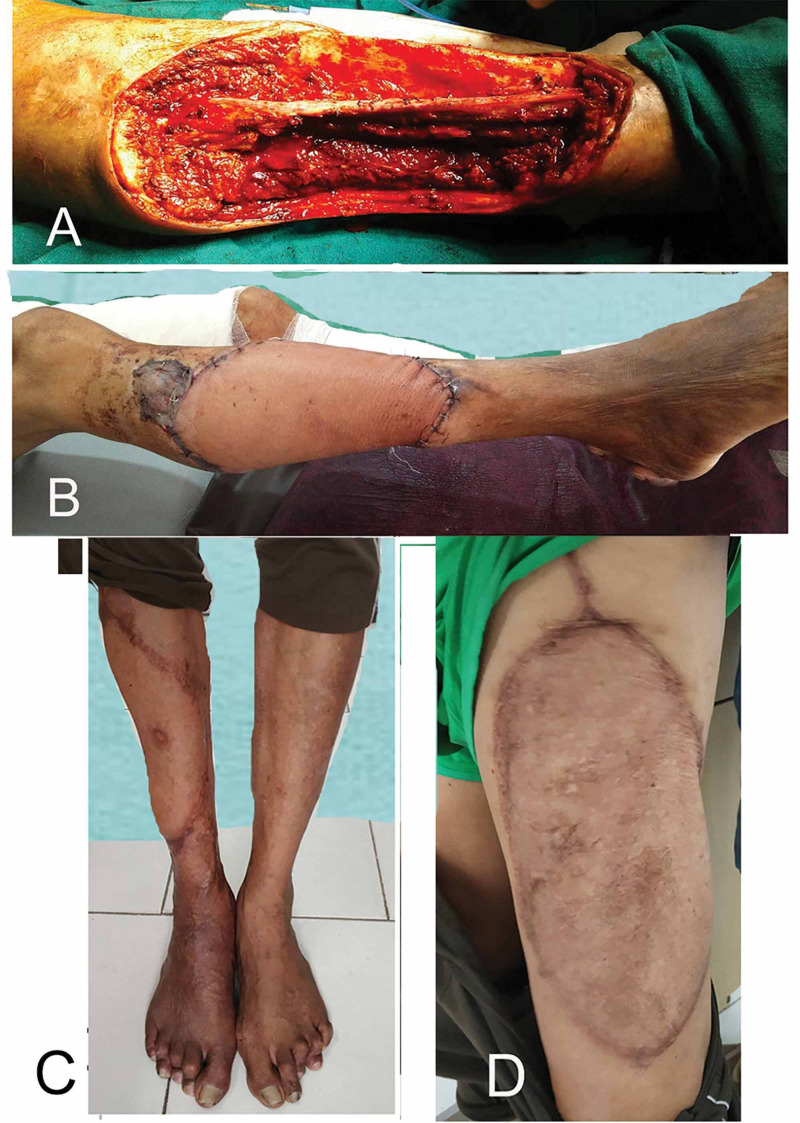
Case 1: Marjolin’s ulcer of the leg with three-year follow-up of flap donor and recipient site. (A) Wide local excision showing exposed tibia, muscles, and the neurovascular structure of the anterior compartment of the leg. (B) Post-operative picture showing free ALT flap and skin graft at the upper margin. (C & D) Three-year follow-up picture of the flap and donor site on the contralateral thigh. ALT, anterolateral thigh.

In our study, nine patients were male and two were female. The mean age of the patients was 54.27 years. On HPE, a well-differentiated SCC was noted in nine patients, and moderately differentiated SCC was noted in two patients. The mean dimensions of ulcer and flap were 8 cm × 6 cm and 18.91 cm × 11 cm, respectively. One of the 11 free ALT flaps was a ‘flow-through’ flap. The posterior tibial artery was used as a recipient vessel in most of the cases (9/11). The femoral and popliteal artery was used in one case each (Figure 1). Inguinal LN dissection was performed in two cases, which came to be negative in both. The flap donor site was skin grafted in all cases (Figure 2 and Table 1).

**Table 1:** Surgical details of free flap surgery. ALT, anterolateral thigh; ILND, inguinal lymph node dissection; LN, lymph node; STSG, split-thickness skin graft

Case no	Age/sex	Location	Pre-operative contracture	Dimension of ulcer	Defect after excision	Treatment	Flap dimension	LN dissection
1	60/M	Leg	60°	10 cm × 8 cm	Exposed tendon and tibia	Free ALT and STSG	28 cm × 17 cm	No
2	30/M	Leg	20°	10 cm × 8 cm	Exposed tendon	Free ALT	20 cm × 10 cm	No
3	55/M	knee	90°	4 cm × 4 cm	Exposed knee joint and femur, perivascular adhesion	Free (flow-through) ALT	25 cm × 15 cm	No
4	71/M	Knee	60°	6 cm × 4 cm	Exposed tendon	Free ALT	15 cm × 8 cm	No
5	38/F	Knee	60°	10 cm × 6 cm	Exposed neurovascular structure and joint	Free ALT	15 cm × 10 cm	ILND
6	60/F	Ankle	20°	4 cm × 4 cm	Exposed tendons	Free ALT	15 cm × 6 cm	No
7	78/M	Thigh	No	10 cm × 10 cm	Exposed bone (femur) and joint	Free ALT	25 cm × 15 cm	ILND
8	48/M	Knee	20°	4 cm × 4 cm	Exposed popliteal artery	Free ALT	15 cm × 10 cm	No
9	52/M	Knee	30°	4 cm × 4 cm	Exposed tendon	Free ALT	15 cm × 10 cm	No
10	45/M	Knee	20°	2 cm × 2 cm	Exposed tendon	Free ALT	15 cm × 10 cm	No
11	60/M	Leg	20°	8 cm × 4 cm	Exposed tendon	Free ALT and STSG	20 cm × 10 cm	No

**Figure 3:**
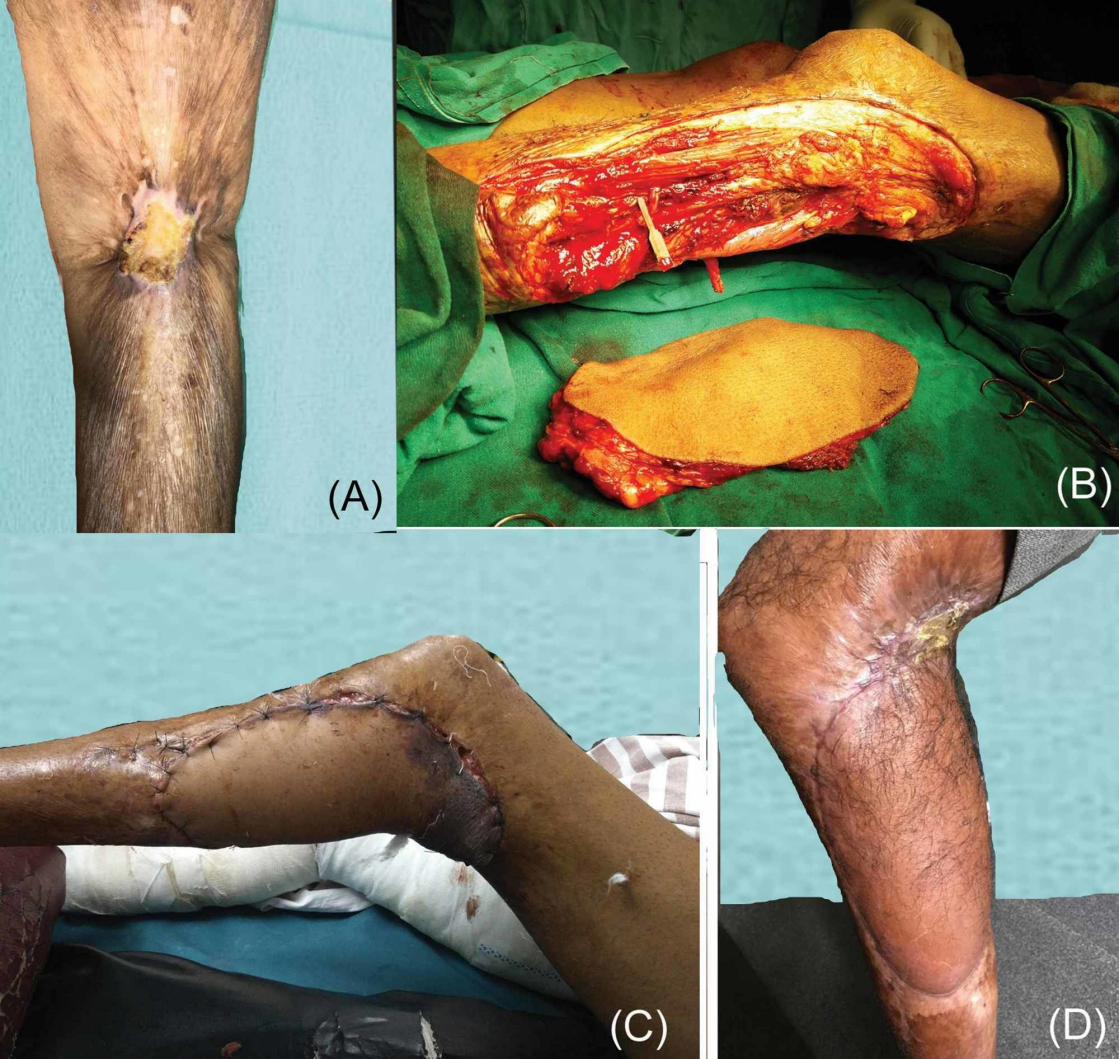
Case 3: Marjolin’s ulcer of the knee joint and upper leg with severe contracture. (A) Ulcerative lesion over the back of the knee. (B) Wide local excision defect of the lesion and harvested (25 cm × 15 cm) ALT flap. (C) First week follow-up picture. Marginal flap necrosis and residual contracture were noted. (D) Residual contracture but well-settled flap. ALT, anterolateral thigh

Flap surgery was successful in all. Marginal flap loss was seen in one case, which was debrided and later skin grafted. The mean follow-up period was 35.82 ± 2.09 months. Partial contracture persisted in two cases within the follow-up period (Figure 3).

Re-contracture was not seen in any of the cases. The limb function was improved in all cases. Complete weight-bearing was achieved in all cases. In five cases, postoperative radiotherapy was given due to a larger size. No recurrence or disease-related mortality was noted within the follow-up period (Table 2).

**Table 2:** Follow-up and outcomes of the surgery.

Case no	Flap-related complications	Radiotherapy	Contracture at 24 months	Limb discrepancy at 24 months	Recurrence	Follow-up period (months)
1	None	Yes	No	No	No	48
2	None	Yes	No	No	No	42
3	Marginal flap loss, debrided and skin grafted	Yes	25°	2 cm	No	42
4	None	No	20°	2 cm	No	Lost in follow-up after 30 months
5	None	Yes	10°	1 cm	No	40
6	None	No	No	No	No	36
7	None	Yes	Arthrodesis	No	No	36
8	None	No	No	No	No	36
9	None	No	No	No	No	Death unrelated to disease after 30 months
10	None	No	No	No	No	30
11	None	No	No	No	No	24

## Discussion

MU is a rare malignancy arising from post-traumatic scar described by French physician Jean-Nicholas Marjolin in 1828. The term ‘Marjolin ulcer’ was coined by Da Costa to describe the malignancy of post-burn scar tissue [10]. Skin breakdown on chronic scars, chronic unhealed ulcers, continuous irritation, secondary intention healing, diminished vascularity, immunity, repeated trauma, and chronic infections of an ulcer are commonly implicated for the development of MU [10-11]. MU of extremities commonly occurs near major joints as these places are subjected to repeated stretching and friction during daily activities. The patients usually present with functional limitations along with ulcers on a deep-seated scar and severe contracture. Extremity is probably the location of the most aggressive form of MU [2-6]. Surgical removal early in the course of the tumor is the mainstay of management, though newer modalities such as cryosurgery, intralesional interferons, 5-fluorouracil or methotrexate, and photodynamic therapy are described without much established definitive role [12]. The outcome of the metastatic disease is very poor, and surgical management and/or chemoradiotherapy have limited benefits other than palliation.

WLE with a 2-cm margin is accepted in most of the recent studies. Resurfacing with a skin graft, local and regional flaps are commonly practiced surgical modalities after the radical excision of MU. Alternatively, an amputation or disarticulation is also a rational approach in advanced MU [3-4,13-14]. The free flap is usually reserved to resurface a larger lesion if a sufficient amount of local tissue is not available.

As per conventional belief, the scar tissue provides a barrier to the spread of the disease. But once the barrier is breached by the ulcer, it is prone to lymphatic spread and distant metastasis. But now it is assumed that malignant cells skip immunological detection by the altered physiological function of the scar tissue. These cells are more prone to metastasis and aggressive malignant transformations [3,15-16]. Metastasis is regarded as the most important prognostic factor [11,15]. In lower limb MU, the ulcer is usually deep-seated at presentation due to repeated cycle of ulcer and secondary intention healing subjecting to persistent searing force on ulcer, and reconstructive need is much beyond the two-dimensional WLE defect. Free flaps are emerging as routine oncoplastic reconstruction for various malignancies. Free latissimus dorsi (LD) flap has been described for reconstruction of MU of scalp earlier [1,10]. Aydogdu et al. also mentioned the use of free flap in their series of surgical reconstruction of MU of different anatomical locations. Though they doubted the radical scar clearance as it removes the protective barrier to the spread of the disease, they recommended the beneficial role of radical tissue clearance for the early-stage disease [17]. Bozkurt et al. recommended the use of free flap in initially skin-grafted and recurrence-free patients as a secondary procedure [18]. Al Maksoud et al. described the use of free LD muscle flap for MU of weight-bearing heel [19]. But, in a background of usually aged and debilitating patient, free flap reconstruction remains much a matter of individual expertise, judgment, and preference.

In our experience, scar contracture in MU is usually deep-seated than the two-dimensional scar and tumor. Due to persistent inflammatory components, the deeper muscle and neurovascular structures are usually seen fibrosed, and three-dimensional WLE excision remains very difficult. Unlike ordinary post-burn contracture, contracture encountered along with MU involves deeper tissue diffusely. It does not have a definite tissue layer of excision and often involves all tissue layers around the neurovascular bundle and tendon. Tendon and neurovascular shortening is not very rare also. High incidence of MU around the knee and vascular shortening is a prominent feature in extremity MU. The reconstructive procedure with other than a free flap could not eliminate contracture optimally, and often amputation remains a safe alternative. The provision of a free flap in pre-operative planning helps an uncompromised radical excision of the lesion as well as scar and contracture tissue. In this way, a free flap improves limb function. Besides, this adds a substantial amount of tissue and reduces residual searing force. As a repeated episode of ulceration and healing due to persistent stretching of scar believes to be prone to malignant degeneration of scar, a free flap may have a beneficial role to prevent re-ulceration of the previous reconstruction, even if it partially covers a large defect. In our cases, the mean dimension of the flap was 18.91 cm × 11 cm, which was approximately 2.59 times larger than the two-dimensional defect after tumor excision with a 2-cm margin. No re-ulceration was noted in a partial free flap and skin graft reconstruction in the cases in our series.

The beneficial role of radiotherapy has been established as an adjuvant treatment in large lesions (>10 cm), margin positive cases, or recurrences by the earlier studies [13-14,17]. As a free flap improves local radiation tolerance than a skin graft reconstruction, an ALT flap adds value to the use of free flap as immediate reconstructive means rather than secondary reconstruction [18]. A free ALT flap does not add much operative morbidity as the whole surgery can be performed only under spinal anesthesia or epidural analgesia in the same sitting, as we have observed in our series.

Free ALT flap also adds its value with a provision of ‘follow-through’ flap, which is popular for limb salvage for vascular injuries in trauma [20,21]. In our study, vascular reconstruction is performed along with resurfacing with such means in one case. The amputation was avoided even after an unexpected intraoperative finding of vascular tethering. Thus, a free ALT flap may be value-added in similar cases.

## Conclusions

Free ALT flap reconstruction of MU of extremity facilitates most radical tumor and scar-contracture removal and thus reduces chances of re-ulceration. It facilitates local radiotherapy protocol with the provision of immediate supple coverage, and thus has a beneficial role other than a secondary reconstructive procedure. Moreover, added benefit may be obtained with a ‘flow-through’ flap to avoid amputation and improve functional outcome.

This was a small case series from a single institute, and outcome analysis on a larger population was beyond the scope due to the infrequent occurrence of MU. Evidence from multiple studies may be necessary to ascertain the role of a free flap for extremity MU reconstruction.
